# Optimizing submerged cultivation of *Bacillus velezensis* for endospore production and formulation

**DOI:** 10.1007/s42770-026-02008-5

**Published:** 2026-07-16

**Authors:** Luana Aparecida Gilio, Gabriel Moura Mascarin, Wagner Bettiol

**Affiliations:** 1 Departamento de Fitopatologia, Universidade Federal de Lavras, Lavras, MG 37200-900 Brazil; 2 Laboratório de Microbiologia Ambiental “Raquel Ghini”, Embrapa Meio Ambiente, Jaguariúna, SP 13.918-110 Brazil

**Keywords:** Culture medium, Fermentation process, Bacterial sporulation, Biological control, Shelf life, Wettable powder

## Abstract

Considering the high efficacy of *Bacillus velezensis* AP-3 strain in controlling plant diseases, optimizing large-scale and cost-effective endospore production is crucial for providing accessible bioproducts to the agricultural sector. This study aimed to optimize the physicochemical and nutritional parameters of *B. velezensis* AP-3 submerged liquid fermentation using cost-effective media suitable for high-yield endospore production. A series of experiments was conducted to evaluate the effects of temperature, ultrasonic wave exposure, inoculum volume, aeration, and culture medium composition on sporulation. Additionally, wettable powder formulations were developed using corn-starch and potato-starch as carriers, supplemented with soybean lecithin. These formulations were assessed for suspensibility and shelf life at room temperature and at 40 °C. Among the physical variables, temperature had the strongest impact on fermentation performance, with endospore production ranging from 4.17 × 10⁷ CFU mL⁻¹ at 22 °C to 7.30 × 10⁸ CFU mL⁻¹ at 34 °C in chemically defined culture medium (T1). Culture medium composition and nutrient sources significantly influenced sporulation. The highest yields were observed in the following media: molasses+cottonseed flour (T8), autoclaved chemically defined culture medium (T1) modified by Vilanova (T6), non-autoclaved chemically defined culture medium (T1) modified by Vilanova (T7), T1 plus sucrose+protein hydrolysate+salts (T11), molasses+protein hydrolysate (T12), molasses+isolated soy protein+yeast extract (T13), and T1 medium modified by Vilanova supplemented with protein hydrolysate (T14). After 72 h of fermentation, these media resulted in yields of 2.32, 2.28, 2.03, 1.17, 1.01, 1.15, and 1.08 × 10⁹ CFU mL⁻¹, respectively. The bio-formulations exhibited a shelf life exceeding 3 years at room temperature.

## Introduction

*Bacilli* are Gram-positive, rod-shaped bacteria belonging to the family *Bacillaceae* within the *Firmicutes* phylum. These bacteria display metabolic flexibility, functioning either as aerobes or facultative anaerobes [1]. Widely distributed, these microorganisms thrive in various ecological niches, including soil, plant surfaces, the rhizosphere, and stored grains [2]. A distinguishing feature of this genus is the production of endospores, which is crucial for the development of commercial *Bacillus*-based products, as this resistant propagule enables formulations with shelf-lives exceeding two years [3].

Endospores are multilayered, highly resistant structures that confer protection against environmental stressors, including UV radiation, desiccation, thermal extremes, and toxic metabolites produced by other organisms [4, 5]. Endospore production is triggered when critical nutrients, such as nitrogen (N), carbon (C), and phosphorus (P), drop below the threshold required for active cell growth [6]. Under favorable environmental conditions, *Bacillus* endospores germinate and express their functional traits, including plant disease control [7] and plant growth promotion [8].

The development of *Bacillus*-based bioproducts containing endospores requires the optimization of fermentation conditions to maximize propagule yield while minimizing production costs (9, 10). Key parameters include physicochemical factors (temperature, aeration, and initial inoculum volume), pH, and nutritional factors (C, N, C: N ratio, salts, and vitamins). These parameters shape not only endospore development but also the generation of secondary metabolites [9–13]. Furthermore, genetic traits play a fundamental role, as they strongly influence the development of endospores and the biosynthesis of secondary metabolites [14].

*Bacillus velezensis* AP-3, previously classified as *Bacillus subtilis* AP-3, is an effective biocontrol agent against numerous plant diseases, including Asian soybean rust caused by *Phakopsora pachyrhizi* [15, 16], coffee leaf rust caused by *Hemileia vastatrix* [17], eucalyptus rust caused by *Puccinia psidii* [18], citrus black spot caused by *Guignardia citricarpa* [19], post-harvest melon rot caused by *Fusarium pallidoroseum* [20], *Fusarium verticillioides* in corn seed [21], and the *Fusarium*–nematode complex in cotton [22], among others. This strain also inhibits mycelial growth of several important phytopathogenic fungi, such as *Rhizoctonia solani*, *Colletotrichum truncatum*, *Sclerotinia sclerotiorum*, *Macrophomina phaseolina*, *Phomopsis* spp., *Bipolaris sorokiniana*, *Pyricularia oryzae*, *Alternaria tenuis*, *Septoria nodorum*, *Sclerotium rolfsii*, *Colletotrichum lindemuthianum*, *Fusarium solani*, and *Fusarium graminearum* [23, 24]. Moreover, *B. velezensis* AP-3 exhibits nematocidal activity against *Meloidogyne* spp. [25], *Heterodera glycines* [26], *Rotylenchulus reniformis*, and *Pratylenchus brachyurus* [27], with performance comparable to chemical nematicides when tested against *Meloidogyne* spp. and *Pratylenchus zeae* [28].

The ability of *B. velezensis* AP-3 to stimulate plant growth has been widely documented [21, 23, 29, 30]. Additionally, this strain has been reported to alleviate the negative effects of salt stress in plants [30]. One of the main mechanisms for controlling plant pathogens is antibiosis, as reported by several authors [17, 22, 31–33]. Beyond AP-3, the biocontrol and plant-growth-promotion potential of other *B. velezensis* strains have been reported [9, 34–39]. Thus, *B. velezensis* is a multifunctional microbe whose application in agriculture should be encouraged.

This study aimed to evaluate and select efficient liquid culture media for endospore production by *B. velezensis* AP-3 through the optimization of nutritional and physicochemical parameters of submerged liquid fermentation. Subsequently, the most promising medium was employed to investigate the growth kinetics of AP-3 strain, followed by development of a wettable powder (WP) formulation based on bacterial endospores and assessment of the shelf life of the final product.

## Materials and methods

All experiments were carried out at the “Raquel Ghini” Environmental Microbiology Laboratory of Embrapa Environment, located in Jaguariúna, São Paulo, Brazil.

### Origin of the isolate, culture maintenance, and inoculum production

The bacterial strain *Bacillus velezensis* AP-3 was originally isolated from rice plants as described by Bettiol [40]. It was initially identified as *Bacillus subtilis* by Bettiol and Kimati [31] and later reclassified as *B. velezensis* by Pacífico et al. [22].

For the initial inoculum, stock cultures were transferred onto Petri dishes (90 × 15 mm) containing nutrient agar (NA) medium (Kasvi, Pinhais, PR, Brazil) and incubated for 48 h at 30 ± 2 °C, in the dark, inside a growth chamber (EL202/4, Eletrolab, São Paulo, SP, Brazil). For subsequent use in this work, 0.8 mL of the bacterial colony suspension was mixed with an equal volume of sterilized 40% (v/v) glycerol solution in cryotubes, resulting in a final concentration of 20%. These bacterial stock cultures were then stored at -20 °C.

In the first experiment, different culture media were evaluated for endospore production, and the inoculum was produced in Luria Bertani (LB) liquid medium (5 g yeast extract; 10 g peptone; 10 g sodium chloride; and 1000 mL distilled water). Liquid cultures were incubated on an orbital shaker (TE-1401, Tecnal^®^, Piracicaba, SP, Brazil) at 250 rpm and 28 °C in the dark for 72 h. After incubation, 1-mL aliquot of the culture was sampled to determine the number of viable cells, expressed in colony-forming units (CFU mL⁻¹).

In the subsequent experiments, the inoculum was produced using Glucose Peptone Yeast (GPY) medium (10 g glucose, 10 g peptone, 5 g yeast extract, 3 g sodium chloride, 1 g monobasic potassium phosphate, 0.5 g magnesium sulfate heptahydrate, 1000 mL water, and pH adjusted to 6) [22]. Growth conditions were the same as described above.

### Determination of colony-forming units (CFU)

To determine the total number of viable cells, the samples were maintained at room temperature prior to analysis. After 35 min, 1 mL of the culture from each Erlenmeyer flask was transferred into a test tube containing 9 mL of diluent solution (9 g of sodium chloride and 1 mL of Tween 80^®^ (Synth, Diadema, SP, Brazil) in 1000 mL of distilled water]. An ultrasonic treatment was applied to the initial dilution using a Unique 1400 A (Unique, Indaiatuba, SP, Brazil) for 5 min. The suspension was agitated three times using an IKA^®^ MS3 Basic Vortex (Merse - Campinas, SP, Brazil), after which serial dilutions were prepared up to 10⁻⁶. From the 10⁻⁵ and 10⁻⁶ dilutions, 100 µL aliquots were plated in triplicate onto Petri dishes containing NA medium and evenly spread over the surface using a Drigalski spatula. After 2 min, the dishes were inverted and incubated at 30 ± 2 °C, in the dark, in a BOD incubator (Solab SL 22, Piracicaba, SP, Brazil). After 24 h of incubation, the number of colony-forming units was recorded (adapted from Bettiol et al. [41]).

For endospore counting, the 10⁻⁵ and 10⁻⁶ dilutions were heated in a water bath at 80 ± 2 °C for 12 min to inactivate vegetative cells. Samples were then subjected to a thermal shock on ice for 10 s, allowed to cool and then plated as previously described. After 24 h of incubation, the number of colony-forming units was enumerated (adapted from Bettiol et al. [41]).

### Medium selection for optimization

Erlenmeyer flasks (250 mL) containing three basal baffles were filled with 90 mL of media T1 to T5 (Table 1) and autoclaved for 20 min at 120 °C. Each flask then received 10 mL of inoculum containing 1 × 10⁸ viable cells mL⁻¹ of *B. velezensis* AP-3. Cultures were incubated in orbital shaker at a constant temperature of 28 ± 2 °C, at 200 rpm, under a 12/12 h light/dark photoperiod, for 72 h. The experiment followed a completely randomized design (CRD), with five treatments and six replicates.

**Table 1:** Composition of the liquid culture media used in the assays to evaluate endospore production by *Bacillus velezensis* AP-3

Culture medium	Composition for 1000 mL of distilled water at pH 7*
T1 - Chemically defined culture medium for bacteria [42]	Glucose – 20 g, KH₂PO₄ – 1 g, MgSO₄·7 H₂O – 0.4 g, CaCl₂·2 H₂O – 0.04 g, FeSO₄·7 H₂O – 0.002 g, ZnSO₄·7 H₂O – 0.0004 g, CuSO₄·5 H₂O – 0.0004 g, MnSO₄·H₂O – 0.0004 g, (NH₄)H₂PO₄ – 3 g, C₆H₅Na₃O₇·2 H₂O – 4 g, Na₂HPO₄ – 37.76 g
T2 - Embrapa liquid medium [2]	Yeast extract – 1 g, meat extract – 3.75 g, peptone – 6.25 g, KH₂PO₄ – 1 g, mineral salt solution – 10 mL (CaCO₃ – 10 g, MgSO₄·7 H₂O – 10 g, FeSO₄·7 H₂O – 1 g, MnSO₄·H₂O – 1 g, ZnSO₄·7 H₂O – 1 g)
T3 - Culture medium for entomopathogenic bacteria (Adapted from Couch [43]	Glucose – 20 g, yeast extract – 2 g, KH₂PO₄ – 1 g, K₂HPO₄ – 1 g, FeSO₄·7 H₂O – 0.02 g, MgSO₄·7 H₂O – 0.3 g, MnSO₄·7 H₂O – 0.02 g, ZnSO₄·7 H₂O – 2 g, (NH₄)₂SO₄ – 2 g, CuSO₄·5 H₂O – 0.005 g, CaCO₃ – 1.25 g, C₁₀H₁₈N₂Na₂O₁₀ – 50 mg
T4 - Complete semi-defined liquid medium (Adapted from Slininger et al. [44]	K₂HPO₄ – 2 g, KH₂PO₄ – 2 g, MgSO₄·7 H₂O – 0.1 g, NaCl – 10 mg, FeSO₄·7 H₂O – 10 mg, ZnSO₄·7 H₂O – 4.4 mg, CaCl₂·2 H₂O – 11 mg, MnCl₂·4 H₂O – 10 mg, (NH₄)₆Mo₇O₂₄·4 H₂O – 2 mg, H₃BO₃ – 2.4 mg, C₁₀H₁₈N₂Na₂O₁₀ – 50 mg. Growth factors: thiamine, riboflavin, calcium pantothenate, niacin, pyridoxamine, thioic acid – 0.5 mg each, and folic acid, biotin, and vitamin B₁₂ – 0.05 mg, Glucose – 40 g, acid hydrolyzed casein – 60 g
T5 - Difco^®^ sporulation medium - DSM [45]	Meat extract – 3 g, peptone – 5 g, KCl – 1 g, MgSO₄ – 0.25 g, Ca(NO₃)₂ 1 M – 1 mL, MnCl₂ 10 mM – 1 mL, and FeSO₄ 1 mM – 1 mL
T6 – T1 medium modified by Vitor Vilanova (autoclaved)	Sucrose – 10 g, isolated soy protein – 4 g, hydrolyzed yeast (Hilyses^®^) – 6 g, MgSO₄ – 0.6 g, KH₂PO₄ – 1 g, NaCl – 2 g, Ca(NO₃)₂ – 1 g, CaCO₃ – 0.7 g, Na₂CO₃ – 0.2 to 0.5 g, silicone-based antifoaming agent – 0.7 mL, ZnSO₄ – 0.01 g, MnSO₄ – 0.02 g, FeSO₄ – 0.04 g, CoSO₄ – 0.005 g, CuSO₄ – 0.005 g
T7 – T6 medium non-autoclaved medium.	T6 plus calcium hypochlorite – 0.015 g
T8 – Molasses + cottonseed flour medium [46]	Molasses – 20 g, cottonseed flour – 10 g
T9 – Bac In^®^medium (Top Bio – Icapuí – Ceará – Brazil)	Commercial medium – 10 g; sucrose – 10 g
T10 – T1 medium + sucrose	T1 with glucose replaced by sucrose – 10 g
T11 – T1 medium + sucrose, protein hydrolysate, and salts	Sucrose – 19 g, KH₂PO₄ – 1 g, MgSO₄·7 H₂O – 0.4 g, CaCl₂·2 H₂O – 0.04 g, FeSO₄·7 H₂O – 0.002 g, ZnSO₄·7 H₂O – 0.0004 g, CuSO₄·5 H₂O – 0.0004 g, MnSO₄·H₂O – 0.0004 g, protein hydrolysate – 8.33 g, C₆H₅Na₃O₇·2 H₂O – 4 g, Na₂HPO₄ – 37.76 g
T12 – Molasses + protein hydrolysate medium	Molasses – 20 g, protein hydrolysate – 10 g
T13 – Molasses + isolated soy protein and yeast medium	Molasses – 20 g, isolated soy protein – 4 g, hydrolyzed yeast Hilyses^®^ – 6 g
T14 – T6 medium + protein hydrolysate	T6 with isolated soy protein replaced by protein hydrolysate – 8.33 g

*The pH of all media was adjusted by using sodium hydroxide or hydrochloric acid solutions (both at 1 mol/L), except for media T6 and T7, which were adjusted by using sodium carbonate. Hydrolyzed yeast Hilyses^®^: (ICC Industrial Comércio Exportação e Importação Ltda.)

### Optimization of physical factors in liquid fermentation for endospore production

The chemically defined medium for bacteria [42], supplemented with 10% (v/v) inoculum containing 1 × 10⁸ viable cells mL⁻¹ of *B. velezensis* AP-3, was selected for physical factor optimization since it demonstrated the highest endospore production according to the previous experiments for evaluation the Medium Selection for Optimization. Each physical factor was evaluated independently using a completely randomized design (CRD), with six replicates per treatment across two independent experiments. All experiments were carried out in 250-mL baffled Erlenmeyer flasks, containing 50 mL of culture medium, incubated for 72 h. The following physical parameters were optimized individually: aeration, temperature, oxygen deprivation stress, inoculum volume, and sonication.

To evaluate the effect of aeration through agitation, flasks were maintained at 150, 200, and 250 rpm using an orbital shaker (Solab SL-223, Piracicaba, Brazil – orbit diameter = 28 mm), and kept at 28 ± 2 °C in the dark. Each flask containing 40.8 mL of culture medium was autoclaved at 121 °C for 20 min, followed by the addition of 4.2 mL of glucose solution (240 g of glucose diluted to a final volume of 1000 mL, i.e. 24%, filled with distilled water and previously sterilized) and 5 mL of inoculum (corresponding to 10% v/v).

In one set of experiments, the effect of incubation temperature was evaluated using shaking baffled flasks maintained at constant temperatures of 22, 28, and 34 °C at 250 rpm in an orbital shaker. All other conditions matched those described for aeration. Since the highest endospore production was achieved at 34 °C, this temperature was selected for all subsequent studies.

On another experiment, to investigate the effect of oxygen deprivation stress, a standard treatment consisting of fermentation with constant agitation for 72 h was compared to treatments in which agitation was interrupted after 24 h, leaving flasks static for 8, 16, or 24 h. Culture flasks were maintained at the same temperature (34 °C) conditions as the other culture flasks. After the static periods, agitation resumed completing the 72-h fermentation time.

To evaluate the effect of inoculum volume on *B. velezensis* growth, four volume rates (0.5%, 1%, 1.5%, and 10% v/v), each calibrated to contain 1 × 10⁸ viable cells mL⁻¹, were tested. Baffled 250-mL Erlenmeyer flasks were filled with 45.55 mL, 45.30 mL, 45.05 mL, or 40.8 mL of liquid medium, respectively, along with glucose (autoclaved solution of 24% glucose) and the corresponding inoculum volume to reach a final volume of 50 mL. Culture flasks were incubated at 34 °C and 250 rpm in the dark on an orbital shaker.

To evaluate the effect of sonication, each Erlenmeyer flask received 45.55 mL of medium, 4.2 mL of glucose, and 250 µL (0.5%) of inoculum. Flasks were incubated at 34 °C, in the dark on an orbital shaker set to 250 rpm. After 4 h of incubation [12], treatments were exposed to ultrasonic baths (Ultrasonic bath with a frequency of 40 kHz) for 10, 20, and 30 min. Afterwards, flasks returned to shaker where they were incubated until fermentation process completed 72 h. Control treatment consisted of uninterrupted fermentation for 72 h.

### Optimization of nutritional factors in liquid fermentation for endospore production

Following the optimization of physical factors of fermentation, the effects of nutritional factors were evaluated to develop a low-cost culture medium capable of yielding at least 1 × 10⁹ CFU mL⁻¹ of endospores.

In comparison to the chemically defined medium adapted from Moraes et al. [42] (T1) (Table 1), four additional culture media were evaluated: T1 medium modified by Vilanova, either autoclaved or not (T6 and T7), molasses + cottonseed flour medium (T8) [46], and the commercial Bac In^®^ medium (T9) ((Top Bio, Icapuí, CE, Brazil) (Table 1). All media were sterilized in an autoclave at 121 °C for 20 min, except for the non-autoclaved medium (T7), which followed the disinfection procedure commonly employed by farmer with calcium hypochlorite. After sterilization or disinfection, carbon sources (sucrose or glucose, as specified in Table 1) were added. Cottonseed flour (Pharmamedia^®^) (ADM Co., Decatur, IL, USA) contains 49.3% C and 9.92% N on a dry weight basis.

The preparation of the non-autoclaved medium (T7 – Villanova’s medium) followed the same procedure routinely applied by the farmer in a system named on-farm production, commonly explored in Brazil. The medium was divided into two beakers. In the first, 100 mL of water, 10 g of sucrose, and 0.015 g L⁻¹ of calcium hypochlorite were dissolved using a magnetic stirrer (Jeio Tech MS-17BY - South Korea) inside a laminar flow hood (Pachane Pa 400 ECO - Piracicaba, SP, Brazil). The remaining ingredients and water were mixed in the second beaker. Once all the ingredients were completely dissolved, the solution from the first beaker was combined with the second and stirred for 3 h. The pH was adjusted to 7 with sodium carbonate, and 49.75 mL of the final medium was transferred to each Erlenmeyer flask. A 0.5% (v/v) inoculum was added, and the flasks were incubated in an orbital shaker at 250 rpm, in the dark, at 34 °C, with six replicates per treatment. After 72 h of incubation, a 1 mL aliquot was collected for endospore quantification (CFU mL⁻¹).

Some of the previously tested culture media were modified in their composition and re-evaluated for endospore production. These media included: the chemically defined medium for bacteria (T1) (42); the molasses + cottonseed meal medium (T8) (46); T1 + sucrose (T10); the sucrose + protein hydrolysate + salts medium (T11); the molasses + protein hydrolysate medium (T12); the molasses + isolated soy protein + yeast medium (T13); and the T6 medium supplemented with protein hydrolysate (T14) (Table 1). Media T10 and T11 were adapted from the chemically defined medium (T1), medium T12 was adapted from T8 while T14 was an adaptation of T6. The pH of all media was adjusted by using sodium hydroxide or hydrochloric acid solutions (both at 1 mol/L), except for media T6 and T7, which were adjusted by using sodium carbonate. Protein hydrolysate contains a minimum of 75% protein, 12 ± 2% total nitrogen. It is composed of 19 amino acids (glutamic acid, leucine, glycine, aspartic acid, proline, serine, valine, arginine, alanine, lysine, phenylalanine, isoleucine, threonine, tyrosine, histidine, methionine, cystine, taurine, and tryptophan) (TransferTech Gestão de Inovação, Erechim, Brazil). The isolated soy protein (Maxsoy, Hortolândia, São Paulo, Brazil) contains 90% protein (~14.4% N).

### Formulation of the wettable powder biocontrol agent

To prepare the formulations, potato starch (Cerealista São José, São Paulo, Brazil) or corn starch (Ingredion^®^, Mogi Guaçu, Brazil) was mixed with the chemically defined medium for bacteria (42) fermented with the *B. velezensis* AP-3 in a 1:1 ratio (w/w). The mixtures were dried in trays inside a drying chamber with forced vertical airflow for 7 days, until the moisture content reached 12% (w/w) for potato starch and 9% for corn starch. Once dried, soybean lecithin (5%) (Quimisul^®^, Joinvile, Brazil) was incorporated in both formulations as a natural surfactant. The powders were then sieved and transferred to sterile cryopreservation tubes, which were kept in dark bags at either room temperature or at 40 °C for shelf-life evaluation. Endospores viability was determined immediately after formulation (deemed time zero), every month for four months, and again after 730 and 1095 days, using three replicates per treatment.

Suspensibility of both formulations was evaluated following the ABNT NBR 13,313 standard [47]. For each test, 2.5 g of the formulation were dispersed in 100 mL of hard water (20 ppm calcium carbonate) at 30 ± 2 °C. After thorough homogenization, the suspension was transferred to a 250-mL graduated cylinder, and the volume adjusted to 250 mL with hard water. The cylinder was sealed and inverted thirty times over a 90-sec period, then allowed to rest for 30 min. Subsequently, a vacuum system was used to remove the supernatant down to the 25 mL mark, and the remaining sediment was transferred to a pre-weighed Petri dish. Samples were dried in an oven (Model 315 SE, Fanem, SP) at 60 ± 2 °C until constant weight. Suspensibility (%) was calculated from the difference between the residue weight and the initial sample weight. Six replicates were evaluated for each formulation.

### Growth kinetics of *B. velezensis* AP-3

The growth curve of *B. velezensis* AP-3 was monitored over five days using six replicates in two selected media: molasses + cottonseed flour (T8) and sucrose + protein hydrolysate + salts (T11) (Table 1). Each medium (99.5 mL), inoculated with 0.5% (v/v) inoculum, was transferred to 250-mL Erlenmeyer flasks with three baffles. The cultures were incubated in an orbital shaker at 250 rpm, maintained at 34 °C, and kept in the dark. At 24-h intervals, a 1-mL aliquot was collected for pH measurement, total cell and viable endospore counts (CFU mL⁻¹).

### Statistical analysis

Data were subjected to the Shapiro-Wilk test to assess the normality of residuals and the Bartlett test to evaluate homogeneity of variances. ANOVA was performed, and means were compared using Tukey’s test at *P* < 0.05. Student’s t test was used to analyse the suspensibility data. All statistical analyses were conducted using RStudio (https://cran.r-project.org/).

## Results

### Culture medium selection for optimization

**Fig. 1:**
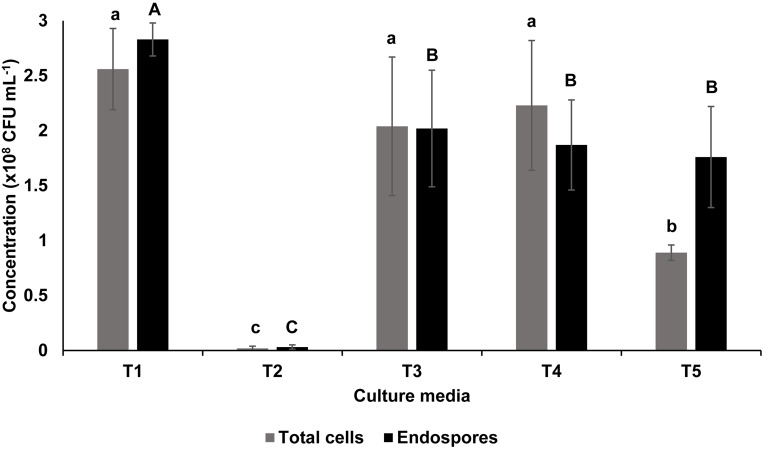
Total cell and endospore production (mean ± standard error) of *Bacillus velezensis* AP-3 expressed in colony-forming units per milliliter (CFU mL⁻¹) under liquid fermentation in the following culture media: T1 – Chemically defined medium for bacteria (Moraes et al. [42]), T2 – Embrapa liquid medium (2), T3 – Culture medium for entomopathogenic bacteria (adapted from Couch [43]), T4 – Complete semi-defined liquid medium (adapted from Slininger et al. [44], and T5 – Difco^®^ sporulation medium – DSM [45]. The composition of the media is described in Table 1. Bars followed by distinct letters indicate significant differences between liquid media, according to Tukey HSD test (*P* < 0.05)

The chemically defined medium for bacteria [42] (T1) resulted in the highest endospore production (2.83 × 10⁸ CFU mL⁻¹). The liquid medium developed by Embrapa (2) (T2), the entomopathogenic bacteria medium by Couch [43] (T3), the modified semi-defined liquid medium by Slininger et al. (44) (T4), and the Difco^®^ endospore formation medium by Nicholson and Setlow [45] (T5) presented yields of 3.10 × 10⁶ CFU mL⁻¹, 2.02 × 10⁸, 1.87 × 10⁸, 1.86 × 10⁸ CFU mL⁻¹, respectively (Fig. 1). Endospore yield obtained in media T3, T4, and T5 did not differ significantly among themselves, but they differed significantly from T1.

When analyzing viable total cell counts, the trends were similar to those observed for endospore production. The chemically defined medium (T1) yielded the highest total cell concentration (2.56 × 10⁸ CFU mL⁻¹), followed by T4 (2.23 × 10⁸ CFU mL⁻¹) and T3 (2.04 × 10⁸ CFU mL⁻¹), which did not differ statistically from one another. However, these media differed from T5 and T2, with cell concentrations of 8.91 × 10^7^ CFU mL⁻¹ and 1.99 × 10^6^ CFU mL⁻¹, respectively (Fig. 1).

### Optimization of physical and nutritional factors in liquid fermentation for endospore production

**Fig. 2:**
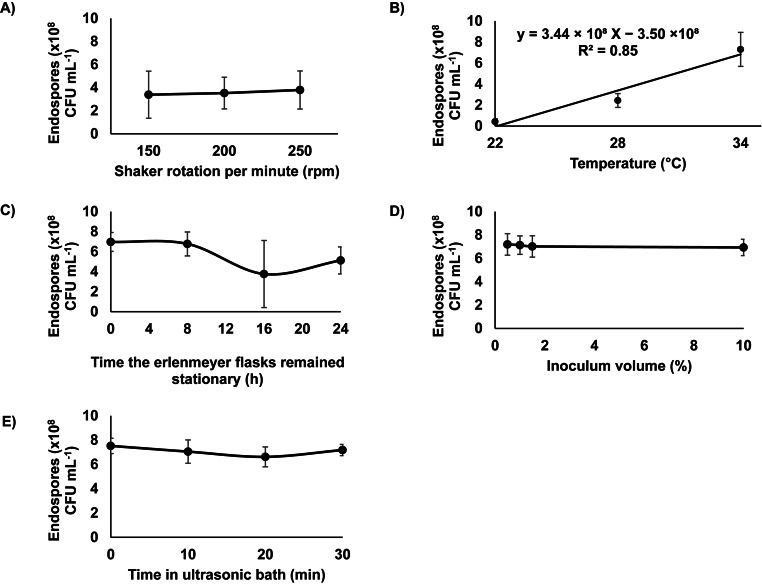
Influence of physical factors on endospore production by *Bacillus velezensis* AP-3, in chemically defined culture medium for bacteria [42] expressed in colony-forming units per milliliter (CFU mL⁻¹). **(a)** Aeration by agitation at 150, 200, and 250 rpm using a shaker; **(b)** Temperature at 22, 28, and 34 °C; **(c)** Aeration under static conditions for 0, 8, 16, and 24 h after 24 h of fermentation, followed by agitation at 250 rpm until completing 72 h; **(d)** Inoculum volume at 0.5, 1.0, 1.5, and 10%; **(e)** Exposure to ultrasonic waves for 0, 10, 20, and 30 min after 4 h of fermentation. Dark circles in the graphs represent mean values with their respective standard errors (SE)

Among the physical factors tested, only temperature influenced significantly endospore production with higher temperatures resulting in increased yields (Fig. 2b). Endospore production reached 7.30 × 10⁸ CFU mL⁻¹ at 34 °C, 2.43 × 10⁸ CFU mL⁻¹ at 28 °C, and 4.17 × 10⁷ CFU mL⁻¹ at 22 °C (Fig. 2b). No significant differences were observed for the other physical factors evaluated (Figs. 2a, c,d, e).

**Fig. 3:**
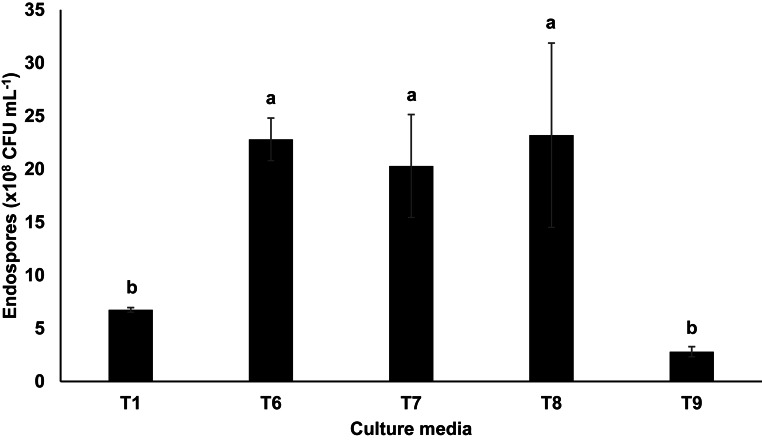
Endospore production by *Bacillus velezensis* AP-3, expressed in colony-forming units per milliliter (CFU mL⁻¹), under liquid-state fermentation using different culture media: T1 – chemically defined medium for bacteria [42]; T6 = T1 medium modified by Vilanova autoclaved; T7 = T6 non-autoclaved; T8 – molasses + cottonseed flour medium [46]; T9 – Bac In^®^ medium. The composition of the culture media is detailed in Table 1. Bars (means ± standard error) followed by distinct letters indicate significant differences between liquid media, according to Tukey HSD test (*P* < 0.05)

In the T6 and T7 media, as well as in the molasses + cottonseed flour medium (T8) [46], endospore production reached 2.28 × 10⁹, 2.03 × 10⁹, and 2.32 × 10⁹ CFU mL⁻¹, respectively, showing no statistical differences among them (Fig. 3). Endospore yields in these three media (T6, T7, and T8) were significantly higher than those obtained in the chemically defined medium [42] (T1) and in the commercial Bac In^®^ medium (T9) commonly used for on-farm production, which yielded 6.75 × 10⁸ and 2.80 × 10⁸ CFU mL⁻¹, respectively (Fig. 3).

**Fig. 4:**
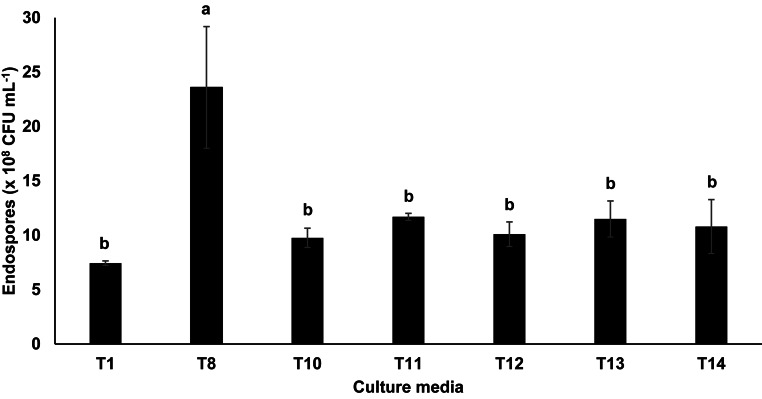
Endospore production by *Bacillus velezensis* AP-3 under liquid-state fermentation using the following culture media: T1 – chemically defined medium for bacteria [42]; T8 – molasses + cottonseed flour medium [46]; T10 = T1 medium + sucrose; T11 = T1 + sucrose + protein hydrolysate + salts medium; T12 – molasses + protein hydrolysate medium; T13 – molasses + isolated soy protein + yeast medium; T14 = T6 medium + with protein hydrolysate. The composition of the media is detailed in Table 1. Bars (means ± standard error) followed by distinct letters indicate significant differences between liquid media, according to Tukey HSD test (*P* < 0.05)

In the study evaluating nutritional modifications, the original medium proposed by Duré et al. [46] (T8) showed the highest endospore production (2.36 × 10⁹ CFU mL⁻¹). The other evaluated media (T1, T10, T11, T12, T13, and T14) (Table 1) did not differ significantly among themselves, with values ranging from 7.44 × 10⁸ to 1.17 × 10⁹ CFU mL⁻¹ (Fig. 4).

### Shelf-life and suspensibility of wettable powder formulations containing *Bacillus velezensis* endospores

**Fig. 5:**
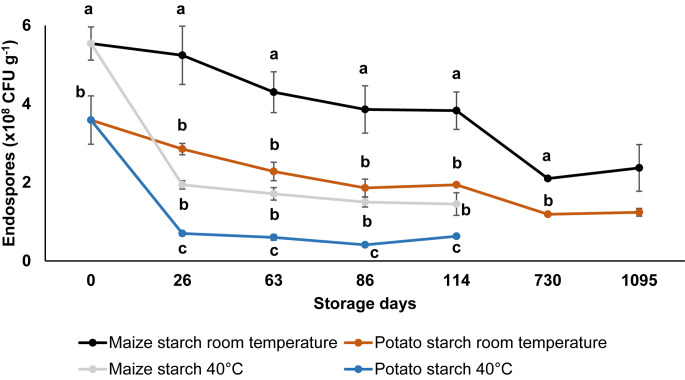
Viability of *Bacillus velezensis* AP-3 endospores formulated with soluble maize starch and potato starch after 26, 63, 86, 114, 730 and 1095 days of storage at room temperature and at 40 °C. The initial concentration of endospores in the culture medium was 4.85 × 10⁸ CFU mL⁻¹ prior to formulation. Curves with circles in the graphs represent mean values with their respective standard errors (SE). Means followed by distinct letters indicate significant differences between formulations, according to Tukey HSD test (*P* < 0.05). The storage stability assessment at 40 °C was terminated on day 114 due to an unforeseen technical failure of the BOD incubator used in the study. As a result, further evaluations under this storage conditions could not be performed beyond this time point

**Fig. 6:**
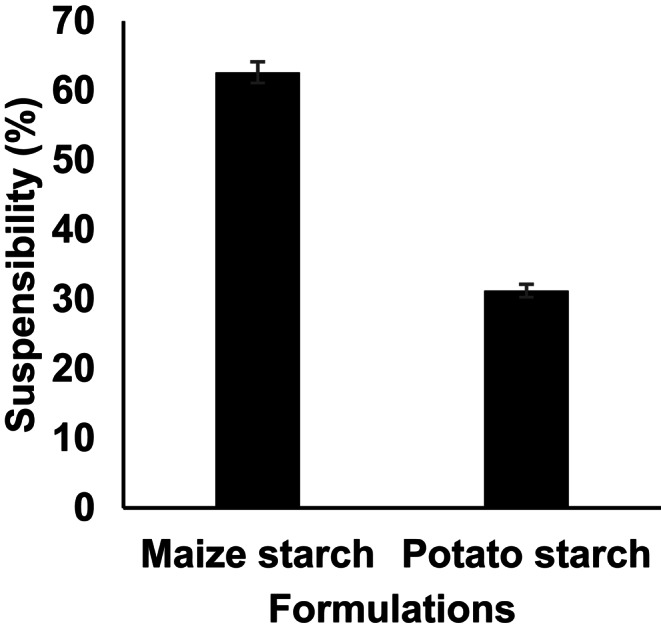
Suspensibility of formulations based on *Bacillus velezensis* AP-3 endospores formulated with corn starch or potato starch as inert carriers and 5% soybean lecithin. The bars represent the treatment means followed by their respective standard errors, according to Student’s t test (*P* < 0.05)

The viability of endospores in formulations using corn starch or potato starch as inert carriers, stored at room temperature, remained stable and viable for up to 1095 days (three years) after formulation (Fig. 5). The mean suspensibility of the corn starch formulation (62.6 ± 2.1) was significantly higher than that of potato starch formulation (31.2 ± 1.8) (*t* = 43.35, df = 8.38, *p* < 0.001, 95% CI: 29.7–33.1) (Fig. 6).

### Growth kinetics of *B. velezensis* AP-3 isolate

**Fig. 7:**
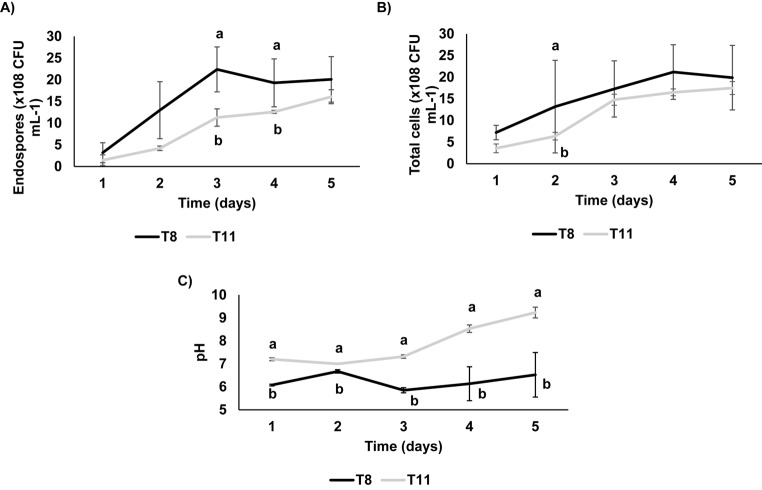
Growth kinetics of *Bacillus velezensis* AP-3 in the molasses + cottonseed flour medium (T8) [46] and in the sucrose + protein hydrolysate + salts medium (T11). The composition of the media is detailed in Table 1. **(a)** Endospore production over the fermentation period (days); **(b)** Total cell production over time; **(c)** Culture medium pH. Lines (mean ± standard error) followed by distinct letters indicate significant differences between endospores, total cells or pH in the two different media according to the Tukey HSD test (*P* < 0.05)

The peak of endospore production occurred on the third day of fermentation in the molasses + cottonseed flour medium (T8) [46], reaching 2.24 × 10⁹ CFU mL⁻¹. In the sucrose + protein hydrolysate + salts medium (T11) endospore production on the third day was 1.13 × 10⁹ CFU mL⁻¹, gradually increasing until the fifth day, reaching 1.61 × 10⁹ CFU mL⁻¹, which corresponds to yields lower than those obtained with the molasses + cottonseed flour medium (T8) (Fig. 7a).

Regarding total cell concentration, the molasses + cottonseed flour medium (T8) [46] peaked with 2.12 × 10⁹ CFU mL⁻¹ at the fourth day. The sucrose + protein hydrolysate + salts medium (T11) showed consistently lower yields throughout the fermentation period, although no significant differences were observed from the second day onward (Fig. 7b).

The pH of the molasses + cottonseed flour medium (T8) [46] remained stable at values close to 6 until the fifth day of fermentation. In the sucrose + protein hydrolysate + salts medium (T11), pH increased throughout fermentation, reaching 9.23 on the fifth day (Fig. 7c).

## Discussion

Endospore production by *Bacillus* species is influenced by multiple factors, including physical, nutritional [9, 48] and genetic [14] variables. In the present study, increasing temperature significantly enhanced endospore production (Fig. 2b), with the highest yield obtained at 34 °C, corresponding to the upper limit of the evaluated range, in the chemically defined bacterial culture medium (T1) described by Moraes et al. [42]. In contrast, agitation rate, inoculum volume, and exposure to ultrasonic waves did not significantly affect endospore production by *B. velezensis* AP-3 (Figs. 2a, c, d, e). Among the 14 culture media evaluated, the molasses + cottonseed flour medium (T8) [46] yielded the highest endospore concentration reaching up to 2.32 × 10⁹ CFU mL⁻¹after only three days of cultivation. Furthermore, both wetable powder (WP) formulations developed in this study, using either potato starch or corn starch as carrier materials, preserved endospore viability for up to 1095 days (3 years) under room-temperature storage conditions (Fig. 5). Collectively, these findings demonstrate the strong influence of physicochemical and nutritional parameters on endospore production by *Bacillus* spp. and highlight the promising industrial potential of *B. velezensis* AP-3 for the development of stable, scalable, and commercially viable bioproducts.

Considering the potential of *Bacillus* in biocontrol of plant diseases, plant growth promotion, and alleviation of abiotic stresses [30, 49], considerable efforts have been made to determine the ideal combination of nutritional and physical conditions for each *Bacillus* isolate [9, 11, 13, 46, 50, 51] aiming to maximize endospore production with batch-to-batch uniformity – an essential requirement for the development of *Bacillus*-based products [52]. In liquid fermentation of *B. velezensis* S26, Russi et al. [9] achieved 10.3 × 10^10^ endospores mL^− 1^, whereas Cao et al. [48] obtained 9.46 × 10⁹ endospores mL^− 1^ optimizing fermentation of *B. velezensis* TCS001. In the present study, the highest endospore yields of *B. velezensis* AP-3 reached up to 2.32 × 10⁹ CFU mL⁻¹, highlighting the importance of fermentation parameters and the genetic potential of each isolate. In mass multiplication of biocontrol agents, several physical parameters may influence cell proliferation, including temperature, aeration, pH, inoculum volume, and inoculum density [9, 11, 12]. In this study, aeration, temperature, inoculum volume, and the effect of ultrasonic waves on endospore production of *B. velezensis* AP-3 were evaluated. Among these factors, only temperature showed a significant positive effect, with 34 °C, the highest temperature tested, resulting in the greatest sporulation (Fig. 2b). This result is consistent with the findings of Russi et al. [9], who reported 37 °C as the optimal temperature for sporulation of *B. velezensis* S26.

Li et al. [12], investigating the effects of ultrasonic waves on biomass production, protease activity, and peptide content in eight *Bacillus* isolates, concluded that exposure of liquid cultures for 10 min in an ultrasonic bath (30 W/L and 20 kHz), one hour after the start of fermentation in a casein-based medium, increased the biomass of *B. velezensis* CICC23571. In contrast, under exposure conditions of 10, 20, or 30 min, 4 h after the beginning of fermentation, no differences in endospore production were observed for *B. velezensis* AP-3 in the present study (Fig. 2e).

Regarding inoculum volume, although high volumes (around 10%) are often employed in optimization studies [9, 53], industrial-scale processes favor smaller volumes (0.5% to 2%) to reduce production costs. In the present study, no significant differences in endospore production were observed across inoculum volumes ranging from 0.5% to 10% (Fig. 2d). This finding was later confirmed in subsequent trials, in which reducing the inoculum volume did not compromise endospore yield.

An important aspect of *Bacillus* fermentation is that the process may occur under strictly aerobic or facultatively anaerobic conditions [1]. In this study, increasing the agitation rate did not significantly affect endospore production (Fig. 2a). This may reflect adequate aeration even at the lower agitation rate (150 rpm), as no supplemental oxygen was supplied, and agitation merely mixed the gaseous and liquid phases within the baffled Erlenmeyer flasks. In a separate trial in which flasks were left at rest for part of the fermentation period, sporulation levels were similar to those of continuously agitated flasks (Fig. 2c). This suggests that the oxygen in the flasks, even at rest, was not fully depleted during this interval.

Both the source and the concentration of C and N in culture media significantly altered endospore production by *B. velezensis* AP-3 (Fig. 4). Cristiano-Fajardo et al. [11] demonstrated that continuous liquid fermentation under carbon (glucose) limitation increased endospore and metabolite production by *Bacillus amyloliquefaciens* 83. Similar results were also reported by Rao et al. [13], Meng et al. [54], and Saberi et al. [10].

Sporulation requires nutrient limitation, since endospore-forming bacteria trigger this mechanism only under specific conditions, generally associated with nutrient depletion and high cell density. In practical terms, to form endospores, bacteria must be in a “starvation state”, characterized by suboptimal nutrient availability [55].

Chemically defined media, in which all constituents and concentrations are precisely known, such as the formulation described by Moraes et al. [42], allow greater control over the physiological and metabolic conditions that regulate bacterial growth and sporulation. In contrast, complex (non-chemically defined) media, typically formulated with plant- or yeast-derived extracts, provide heterogenous nutrient compositions that may better mimic natural environmental conditions and, consequently, stimulate sporulation more effectively. Nevertheless, the intrinsic compositional variability of these substrates may lead to batch-to-batch inconsistencies and reduced process reproducibility. Therefore, the selection of an appropriate fermentation medium should carefully balance the specific objectives of the study with the level of standardization and consistency required for reliable and scalable endospore production.

The incorporation of agro-industrial by-products into complex fermentation media represents an important strategy to reduce production costs while providing essential nutrients required for vegetative growth and endospore formation, thereby improving both the sustainability and economic competitiveness of the bioprocess. Examples of such low-cost substrates include dried brewer’s yeast, soybean meal, sucrose, fructose, skim milk, malt extract, protein hydrolysates, apple concentrate, whey, sugarcane and beet molasses, yeast extracts, peptone, and meat-, fish-, or bone-derived meals [56]. Moreover, the remarkable metabolic versatility of *B. velezensis*, including its capacity to assimilate diverse organic and inorganic carbon and nitrogen sources and to tolerate broad ranges of pH, osmotic pressure, temperature, and aeration, further highlights its suitability for industrial-scale fermentation. Collectively, these physiological and technological attributes reinforce the strong potential of *B. velezensis* as a commercially viable biocontrol agent for large-scale production and global agricultural applications.

The chemically defined culture medium (T1) [42], modified by Vilanova, and either autoclaved (T6) or chemically disinfected with calcium hypochlorite as the sterilizing agent (T7), showed no statistical difference when compared to the molasses + cottonseed flour medium (T8) [46], which numerically produced the highest endospore yields (Fig. 3). Notably, T6 and T7 media used lower-cost ingredients. Another relevant finding was the absence of visible contamination on Petri dishes when the medium was disinfected with calcium hypochlorite instead of autoclaving. However, further studies using additional methods are needed to confirm microbiological safety and detect possible contaminants. Calcium hypochlorite was selected for chemical disinfection as it reflects the current practice among local farmers in Brazil. Nevertheless, prior to issuing a definitive technical recommendation, further microbiological analyses will be conducted in future studies by our group, given that such evaluation was beyond the scope of this study.

Sodium and calcium hypochlorite are sanitizers widely used for cleaning and disinfecting milking equipment, reducing the spread of mastitis-causing microorganisms. Sodium hypochlorite is especially recommended for sanitizing fruits and vegetables due to its accessibility, low cost, and satisfactory efficiency [57]. Gavilan et al. [58] demonstrated that exposure to 1.5% sodium hypochlorite for 30 min effectively sterilized the culture medium and ensured the survival and development of *Cochlospermum regium*, highlighting its potential for reducing production costs. Similarly, Cardoso and Imthurn [59] reported that chlorine dioxide (25 mg L^− 1^) led to low contamination rates and healthy growth of *Gerbera jamesonii*, without altering the physicochemical quality of the medium. These results reinforce chemical sterilization as a practical, accessible, and technically viable method for on-farm microbial multiplication. However, before any generalized recommendation, additional studies must be conducted considering regulatory and quality control restrictions.

Formulations of biocontrol agents are essential to ensure shelf-life and facilitate field application [60]. Vignesh et al. [61] formulated *B. velezensis* in tablet form, achieving a 180-day shelf-life at room temperature, with endospore counts decreasing from 5.3 × 10⁸ to 2.3 × 10⁸ during this period. Chumthong et al. [62] formulated water-soluble granules (WSG) of *Bacillus megaterium* endospores using sodium alginate, lactose, and polyvinylpyrrolidone (PVP K-30), maintaining viability for 24 months at room temperature (counts decreasing from 3.75 ± 0.75 × 10⁹ CFU g^− 1^ to 1.67 ± 0.65 × 10⁹ CFU g^− 1^).

In this study, *B. velezensis* AP-3 endospores formulated as WP using either corn starch or potato starch as carrier materials maintained high viability for up to 1095 days (3 years) under room-temperature storage conditions (Fig. 5). In the corn starch-based formulation, viability decreased only from 5.54 × 10⁸ to 2.37 × 10⁸ CFU g^− 1^ over the storage period, demonstrating remarkable long-term stability. In contrast, storage at 40 °C caused a pronounced and accelerated decline in viability in both formulations compared with those maintained at room temperature (Fig. 5). These findings emphasize the critical role of appropriate storage conditions in preserving the quality, stability, and efficacy of endospore-based bioproducts, particularly by minimizing temperature-associated losses during long-term storage and commercialization.

Demand for sustainable and efficient biological products is constantly increasing worldwide. *Bacillus velezensis* exhibits low environmental impact and has been explored as a probiotic in animal health [63], soil decontaminant [64], and biocontrol agent of plant diseases. The results obtained in this study, together with previous findings on *B. velezensis* AP-3 [15–32], reinforce the feasibility of developing stable and economically viable endospore-based formulations for multiple agricultural applications. In addition, the high resistance and shelf stability of bacterial endospores facilitate downstream processing, long-term storage, transportation, and formulation under commercial conditions, which are critical parameters for large-scale bioproduct manufacturing. The use of low-cost nutrient sources and scalable fermentation conditions further supports the economic viability of industrial production. Future studies should therefore focus on pilot- and industrial-scale validation of the fermentation process, optimization of process parameters under large-volume cultivation, and development of robust formulations for field evaluation and commercial deployment.

## Data Availability

Data are available upon request.

## References

[1] Ajilogba CF, Babalola OO (2013) Integrated management strategies for tomato *Fusarium* wilt. Biocontrol Sci 18:117–127. 10.4265/bio.18.117 24077535 10.4265/bio.18.117

[2] Monnerat R, Montalvão SCL, Martins ES, Queiroz PRM, Silva EYY, Garcia ARM, Castro MT, Rocha GT, Ferreira ADCL, Gomes ACMM (2020) Manual de produção e controle de qualidade de produtos biológicos à base de bactérias do gênero *Bacillus* para uso na agricultura. Embrapa Recursos Genéticos e Biotecnologia. www.infoteca.cnptia.embrapa.br/infoteca/handle/doc/1122563. Accessed 15 June 2022

[3] Galindo E, Serrano Carreón L, Gutiérrez CR, Allende R, Balderas K, Patiño M, Trejo M, Wong MA, Rayo E, Isauro D, Jurado C (2013) The challenges of introducing a new biofungicide to the market: A case study. Elet J Biotechnol 16:1–23. 10.2225/vol16-issue3-fulltext-6

[4] Higgins D, Dworkin J (2012) Recent progress in *Bacillus subtilis* sporulation. FEMS Microbiol Rev 36:131–148. 10.1111/j.1574-6976.2011.00310.x 22091839 10.1111/j.1574-6976.2011.00310.x PMC3237856

[5] Lyngwi NA, Joshi SR (2014) Economically important *Bacillus* and related genera: a mini review. In: Arnab S (ed) Biology of useful plants and microbes. Narosa Publishing House, New Delhi, pp 33–43

[6] Earl AM, Losick R, Kolter R (2008) Ecology and genomics of *Bacillus subtilis*. Trends Microbiol 16:269–275. 10.1016/j.tim.2008.03.004 18467096 10.1016/j.tim.2008.03.004 PMC2819312

[7] Dutilloy E, Arias AA, Richet N, Guise J, Duban M, Leclere V, Selim S, Jacques P, Jacquard C, Clément C, Barka EA, Esmaeel Q (2024) *Bacillus velezensis* BE2 controls wheat and barley diseases by direct antagonism and induced systemic resistance. Appl Microbiol Biotechnol 108:64. 10.1007/s00253-023-12864-y 38189957 10.1007/s00253-023-12864-y PMC13496951

[8] Nunes PSO, Medeiros FHV, Oliveira TS, Zago JRA, Bettiol W (2022) *Bacillus subtilis* and *Bacillus licheniformis* promote tomato growth. Braz J Microbiol 54:397–406. 10.1007/s42770-022-00874-3 36422850 10.1007/s42770-022-00874-3 PMC9943921

[9] Russi A, Granada CE, Schwambach J (2024) Optimization of *Bacillus velezensis* S26 sporulation for enhanced biocontrol of gray mold and anthracnose in postharvest strawberries. Postharvest Biol Technol 210:112737. 10.1016/j.postharvbio.2023.112737

[10] Saberi F, Marzban R, Ardjmand M, Shariati FP, Tavakoli O (2020) Optimization of culture media to enhance the ability of local *Bacillus thuringiensis* var. *tenebrionis*. J Saudi Soc Agric Sci 19:468–475. 10.1016/j.jssas.2020.08.004

[11] Cristiano-Fajardo AS, Flores C, Flores N, Tinoco-Valencia R, Serrano-Carreón L, Galindo E (2019) Glucose limitation and glucose uptake rate determines metabolite production and sporulation in high cell density continuous cultures of *Bacillus amyloliquefaciens* 83. J Biotechnol 299:57–65. 10.1016/j.jbiotec.2019.04.027 31055146 10.1016/j.jbiotec.2019.04.027

[12] Li Y, Ruan S, Zhou A, Xie P, Azam SMR, Ma H (2022) Ultrasonic modification on fermentation characteristics of *Bacillus* varieties: Impact on protease activity, peptide content and its correlation coefficient. LWT - Food Sci Technol 154:112852. 10.1016/j.lwt.2021.112852

[13] Rao YK, Tsay K, Wu W, Tzeng Y (2007) Medium optimization of carbon and nitrogen sources for the production of spores from *Bacillus amyloliquefaciens* B128 using response surface methodology. Process Biochem 42:535–541. 10.1016/j.procbio.2006.10.007

[14] Ruiz-Garcia C, Béjar V, Martínez-Checa F, Llamas I, Quesada E (2005) *Bacillus velezensis* sp. nov., a surfactant-producing bacterium isolated from the river Velez in Malaga, southern Spain. Int J Syst Evolut Microbiol 55:191–195. 10.1099/ijs.0.63310-0 10.1099/ijs.0.63310-0 15653875

[15] Dorighello DV, Bettiol W, Maia NB, Leite RMVBC (2015) Controlling Asian soybean rust (*Phakosora pachyrhizi*) with *Bacillus* spp. and coffee oil. Crop Prot 67:59–65. 10.1016/j.cropro.2014.09.017

[16] Dorighello DV, Forner C, Leite RMVBC, Bettiol W (2020) Management of Asian soybean rust with *Bacillus subtilis* in sequential and alternating fungicide applications. Aust Plant Pathol 49:79–86. 10.1007/s13313-019-00677-5

[17] Bettiol W, Varzea VMP (1992) Controle biológico da ferrugem (*Hemileia vastatrix*) do cafeeiro com *Bacillus subtilis* em condições controladas. Fitopatol Bras 17:91–95

[18] Santos CCF, Castro HA, Bettiol W, Júnior AA (1998) Sensibilidade *in vitro* de urediniósporos de *Puccinia psidii* a *Bacillus subtilis*. Summa Phytopathol 24:183–118

[19] Bernardo ERA, Bettiol W (2010) Controle da pinta preta dos frutos cítricos em cultivo orgânico com agentes de biocontrole e produtos alternativos. Trop Plant Pathol 35:37–42. 10.1590/S1982-56762010000100006

[20] Terao D, Forner C, Maia AHN, Bettiol W (2014) Potential use of bioagents in the control of postharvest rot in melon. Acta Horticult 1053:65–74. 10.17660/ActaHortic.2014.1053.4

[21] Ferreira TC, Lago L, Silva LG, Pacifico MG, Faria MR, Bettiol W (2021) Potencial de *Bacillus* spp. em promover o crescimento e controlar *Fusarium verticillioides* em milho. Summa Phytopathol 47:195–203. 10.1590/0100-5405/241384

[22] Pacífico MG, Eckstein B, Bettiol W (2021) Screening of *Bacillus* for the development of bioprotectans for the control of *Fusarium oxysporum* f. sp. vasinfectum Meloidogyne incognita Biol Control 164:104764. 10.1016/j.biocontrol.2021.104764

[23] Araújo FF, Henning AA, Hungria M (2005) Phytohormones and antibiotics produced by *Bacillus subtilis* and their effects on seed pathogenic fungi and on soybean root development. World J Microbiol Biotechnol 21:1639–1645. 10.1007/s11274-005-3621-x

[24] Lazzaretti E, Menten JOM, Bettiol W (1994) *Bacillus subtilis* antagônicos aos principais patógenos associados a sementes de feijão e trigo. Fitopatol Venez 7:42–46

[25] Mazzuchelli RCL, Araújo FF (2011) Eficácia do controle de nematoides por *Bacillus subtilis* em duas variedades de cana-de-açúcar. Colloq Agrar 7:51–58

[26] Araújo FF, Silva JFV, Araújo ASF (2002) Influência de *Bacillus subtilis* na eclosão, orientação e infecção de *Heterodera glycines* em soja. Ciênc Rural 32:197–202. 10.1590/S0103-84782002000200003

[27] Higaki WA (2012) *Bacillus subtilis* e abamectina no controle de *Rotylenchulus reniformis* e *Pratylenchus brachyurus* e alterações fisiológicas em algodoeiro em condições controladas. Dissertation, Universidade do Oeste Paulista

[28] Morgado TDT, Guerra JT, ber FF, Mazzuchelli RCL (2015) Effectiveness and persistence of biological control of nematodes in sugarcane. Afr J Agric Res 10:4490–4495. 10.5897/AJAR2015.10344

[29] Cardozo RB, Araújo FF (2011) Multiplicação de *Bacillus subtilis* em vinhaça e viabilidade no controle da meloidoginose, em cana-de-açúcar. Rev Bras Eng Agr Ambl 15:1283–1288. 10.1590/S1415-43662011001200010

[30] Medeiros CAA, Bettiol W (2021) Multifaceted intervention of *Bacillus* spp. against salinity stress and *Fusarium* wilt in tomato. J Appl Microbiol 131:2387–2401. 10.1111/jam.15095 33817910 10.1111/jam.15095

[31] Bettiol W, Kimati H (1989) Seleção de microrganismos antagônicos a *Pyricularia oryzae* Cav. para o controle da brusone do arroz (*Oryza sativa* L). Summa Phytopathol 15:257–266

[32] Bettiol W, Kimati H (1990) Efeito de *Bacillus subtilis* sobre *Pyricularia oryzae* agente causal da brusone do arroz. Pesq Agropecu Bras 25:1165–1174

[33] Bettiol W, Saito ML, Brandão MSB (1994) Controle da ferrugem do cafeeiro com produtos à base de *Bacillus subtilis*. Summa Phytopathol 20:119–123

[34] Bo-Eun Kim H, Won M, Jeong M, Oh K, Park DS (2023) Characterization and genomic insight of surfactin producing *Bacillus velezensis* and its biocontrol potential against pathogenic contamination in lettuce hydroponics. Environ Sci Pol Res 30:121487–121500. 10.1007/s11356-023-30871-4 10.1007/s11356-023-30871-4 37950785

[35] Fan Y, He X, Dai J, Yang N, Jiang Q, Xu Z, Tang X, Yu Y, Xiao M (2023) Induced resistance mechanism of *Bacillus velezensis* S3 1 against pepper wilt. Cur Microbiol 80:367. 10.1007/s00284-023-03470-2 10.1007/s00284-023-03470-2 37819393

[36] Yao Y, Wang L, Zhai H, Dong H, Wang J, Zhao Z, Xu Y (2025) *Bacillus velezensis* A-27 as a potential biocontrol agent against *Meloidogyne incognita* and effects on rhizosphere communities of celery in field. Sci Rep 15:1057. 10.1038/s41598-024-83687-8 39774715 10.1038/s41598-024-83687-8 PMC11707364

[37] Wang Q, Sun Z, Li T, Fan T, Zhou Z, Liu J, Chen X, Wang A (2025) Identifying a biocontrol bacterium with disease-prevention potential and employing it as a powerful biocontrol agent against *Fusarium oxysporum*. Int J Molec Sci 26:700. 10.3390/ijms26020700 39859414 10.3390/ijms26020700 PMC11766301

[38] Peng T, Qu F, Wang Z, Li Q, Wang X, Zhang Y, Xiong X, Li G, Hu S, Hu X (2025) *Bacillus velezensis* enhances tomato seedling productivity by coordinating functional traits and substrate properties systematically. Sci Horticult 339:113905. 10.1016/j.scienta.2024.113905

[39] Russi A, Granada CE, Schwambach J (2025) Antagonistic potential of *Bacillus velezensis* S26 endospores against gray mold in grapevines. J Plant Dis Prot 132:2. 10.1007/s41348-024-01040-7

[40] Bettiol W (1988) Seleção de microrganismos antagônicos a *Pyricularia oryza*e CAV. para o controle da brusone do arroz *(Ory*z*a sati*va L.). Thesis, Universidade de São Paulo

[41] Bettiol W, Morandi MAB, Pinto ZV, Lucon CMM (2022) Controle de qualidade e conformidade de produtos e fermentados à base de *Bacillus* spp.: proposta metodológica. Embrapa Meio Ambiente, Jaguariúna. www.infoteca.cnptia.embrapa.br/infoteca/handle/doc/1146275. Accessed 17 January 2023

[42] Moraes IO, Capalbo DMF, Moraes RO (1991) Multiplicação de agentes de controle biológico. In: Bettiol W (ed) Controle Biológico de doenças de plantas. Embrapa, Jaguariúna pp 253–272

[43] Couch TL (2000) Industrial fermentation and formulation of entomopathogenic bacteria. In: Charles JF (ed) Entomopathogenic bacteria: from laboratory to field applications. Kluwer Academic, New York, pp 297–316. 10.1007/978-94-017-1429-7_16

[44] Slininger PJ, Schisler DA, Ericsson LD, Brandt TL, Frazier MJ, Woodell LK, Olsen NL, Kleinkopf GE (2007) Biological control of post-harvest late blight of potatoes. Biocontrol Sci Technol 17:647–663. 10.1080/09583150701408881

[45] Nicholson WL, Setlow P (1990) Sporulation, germination and outgrowth. In: Harwood CR, Cutting SM (eds) Molecular biological methods for *Bacillus*. John Wiley & Sons Ltd, Chichester, pp 391–450

[46] Duré LMM, Mascarin GM, Bettiol W (2025) Optimization of endospore production by solid and liquid fermentation for the development of effective formulations of *Bacillus velezensis*-based products. Braz J Microbiol 56:1253–1261. 10.1007/s42770-025-01626-9 39961998 10.1007/s42770-025-01626-9 PMC12095093

[47] ABNT – Associação Brasileira de Normas Técnicas (2000) ABNT NBR 13313: agrotóxico: determinação da suspensibilidade. Rio de Janeiro

[48] Cao H, Chen Z, Li X, Song G, Wu Y, Jin J, Cui F, Yuan J, Qi H, Wang J, Chen J (2024) Optimization of fermentation conditions for *Bacillus velezensis* TCS001 and evaluation of its growth promotion and disease prevention effects on strawberries. Biol Control 198:105632. 10.1016/j.biocontrol.2024.105632

[49] Fonseca MC, Bossolani JW, Oliveira SL, Moretti LG, Portugal JR, Scudeletti D, Oliveira EF, Crusciol CAC (2022) *Bacillus subtilis* inoculation improves nutrient uptake and physiological activity in sugarcane under drought stress. Microorganisms 10:809. 10.3390/microorganisms10040809 35456859 10.3390/microorganisms10040809 PMC9029642

[50] Lima-Pérez J, López-Pérez M, Viniegra-González G, Loera O (2019) Solid-state fermentation of *Bacillus thuringiensis* var *kurstaki* HD-73 maintains higher biomass and spore yields as compared to submerged fermentation using the same media. Bioproc Biosyst Engin 42:1527–1535. 10.1007/s00449-019-02150-5 10.1007/s00449-019-02150-5 31115662

[51] Monteiro SM, Clemente JJ, Henriques AO, Gomes RJ, Carrondo MJ, Cunha AE (2005) A procedure for high-yield spore production by *Bacillus subtilis*. Biotechnolo Progr 21:1026–1031. 10.1021/bp050062z 10.1021/bp050062z 16080679

[52] Posada-Uribe LF, Romero-Tabarez M, Villegas-Escobar V (2015) Effect of medium components and culture conditions in *Bacillus subtilis* EA-CB0575 spore production. Bioproc Biosyst Engin 38:1879–1888. 10.1007/s00449-015-1428-1 10.1007/s00449-015-1428-1 26135004

[53] Wen J, Pi N, He F, Zeng Y, Weng Q, Luo J (2025) Optimization of the fermentation and preparation of the wettable powder formulation of *Bacillus velezensis* F0b. Microorganisms 13:560. 10.3390/microorganisms13030560 40142453 10.3390/microorganisms13030560 PMC11944301

[54] Meng F, Zhu X, Zhao H, Lu F, Lu Y, Lu Z (2020) Improve production of pullulanase of *Bacillus subtilis* in batch and fed-batch cultures. Appl Biochem Biotechnol 193:296–306. 10.1007/s12010-020-03419-2 32954482 10.1007/s12010-020-03419-2

[55] Errington J (2003) Regulation of endospore formation in *Bacillus subtilis*. Nat Rev Microbiol 1:117–126. 10.1038/nrmicro750 15035041 10.1038/nrmicro750

[56] Costa E, Teixidó N, Usall J, Atarés E, Viñas I (2001) Production of the biocontrol agent *Pantoea agglomerans* strain CPA-2 using commercial products and by-products. Appl Microbiol Biotechnol 56:367–371. 10.1007/s002530100666 11549003 10.1007/s002530100666

[57] Ribeiro JM, Canuto KM, Veschi JLA (2008) Compostos clorados: aspectos gerais e sua utilização como agente sanitizante na agricultura, micropropagação e pecuária. Embrapa Semi-Árido. www.infoteca.cnptia.embrapa.br/infoteca/handle/doc/159162. Accessed 20 December 2024

[58] Gavilan NH, Furlan FC, Zorz AZ, Oliveira LS, Campos WF, Brondani GE (2018) Chemical sterilization of culture medium for *in vitro* multiplication of *Cochlospermum regium*. Ciênc Rural 48:e20170581. 10.1590/0103-8478cr20170581

[59] Cardoso JC, Imthurn ACP (2018) Easy and efficient chemical sterilization of the culture medium for in vitro growth of gerbera using chlorine dioxide (ClO_2_). Ornaml Hortic 24:218–224. 10.14295/oh.v24i3.1222

[60] Teixidó N, Usall J, Torres R (2022) Insight into a successful development of biocontrol agents: production, formulation, packaging, and shelf life as key Aspects. Horticulture 8:305. 10.3390/horticulturae8040305

[61] Vignesh M, Shankar SM, Subramani N, VedhaHari BN, Ramyadevi D (2022) Study on spray-drying of *Bacillus velezensis* NKMV-3 strain, its formulation and bio efficacy against early blight of tomato. Biocat Agric Biotechol 45:102483. 10.1016/j.bcab.2022.102483

[62] Chumthong A, Kanjanamaneesathian M, Pengnoo A, Wiwattanapatapee R (2008) Water-soluble granules containing *Bacillus megaterium* for biological control of rice sheath blight: formulation, bacterial viability and efficacy testing. World J Microbiol Biotechnol 24:2499–2507. 10.1007/s11274-008-9774-7

[63] Chen B, Zhou Y, Duan L, Gong X, Liu X, Pan K, Zeng D, Ni X, Zeng Y (2023) Complete genome analysis of *Bacillus velezensis* TS5 and its potential as a probiotic strain in mice. Front Microbiol 14:1322910. 10.3389/fmicb.2023.1322910 38125573 10.3389/fmicb.2023.1322910 PMC10731255

[64] Zhang C, Chen L, Si H, Gao W, Liu P, Zhang J (2020) Study on the characteristics and mechanisms of nicosulfuron biodegradation by *Bacillus velezensis* CF57. J Basic Microbiol 60:649–658. 10.1002/jobm.202000039 32378242 10.1002/jobm.202000039

